# COVID-19 randomised trial protocols: rapid publication without barriers

**DOI:** 10.1186/s13063-020-04304-3

**Published:** 2020-04-15

**Authors:** Shaun Treweek, Peter Jüni, Tianjing Li, Jonathan Collin, Matthias Briel, An-Wen Chan, Karla Hemming, William J. Meurer, Matthew R. Sydes, Jeremy M. Grimshaw

**Affiliations:** 1 University of Aberdeen, Aberdeen, UK; 2 University of Toronto, Toronto, Canada; 3 University of Colorado Denver, Denver, USA; 4 BMC, London, UK; 5 University of Basel, Basel, Switzerland; 6 University of Birmingham, Birmingham, UK; 7 University of Michigan, Ann Arbor, USA; 8 University College London, London, UK; 9 Ottawa Health Research Institute, Ottawa, Canada

**Keywords:** COVID-19

We are living in extraordinary times. COVID-19 is having a profound effect on every aspect of our lives. Most who read this will do so from their own homes. It will have been weeks since many of us were at our normal place of work, and it is likely to be many weeks, probably months, before we are back there. Videoconferencing is becoming second nature for us all.

Research into COVID-19 is exploding, as it needs to, and this includes randomised trials. Between 23 January 2020 and 8 March 2020, there were 382 new registered COVID-19 trials on the World Health Organisation International Clinical Trials Registry Platform (ICTRP) [1]. The ICTRP COVID-19 trial list had grown to 586 by 31 March 2020 [2]. Most are based in China, but the COVID-19 pandemic is now global, and trials are moving with it. Governments and the public are clamouring for safe and effective treatments. Without rigorous, prospective, randomised trials, we risk exposing many people to treatments that might not work. Now more than ever, we need trialists to predefine and publicly post their designs and analyses.

For transparency, for clear reporting, and to avoid duplication of effort, researchers need to know details about these trials, and they need to know quickly. *Trials* is proud of its record of publishing trial protocols, but the process can be time-consuming in normal times, and these are far from normal times.

*Trials* wants to make publishing protocols for COVID-19 trials that have ethical approval much faster and much simpler. Starting immediately, *Trials* is implementing a simplified process for handling COVID-19 trial protocols. We understand that funding may be limited or difficult to secure at this time. BMC, the publisher of *Trials*, has a robust commitment to ensuring that all work is published without barriers for authors who may lack funding, especially important during the COVID-19 outbreak. If you anticipate difficulty in speedily covering the cost of article-processing charges, please state this in your initial inquiry and request a fee waiver. The process is outlined in Table 1.

**Table 1:** New handling process for COVID-19 trial protocols

What authors do	What we do
Visit https://trialsjournal.biomedcentral.com/covid-19 [3]. Download the structured summary and follow the links to contact us stating your interest in submitting a COVID-19 structured summary and protocol. The trial must already have ethical approval. Please also state if you anticipate difficulty in covering the article-processing charge.	We send instructions and feedback for rapid submission. Every effort will be made to respond within 24 h.
Submit structured summary to Editorial Manager (https://www.editorialmanager.com/trls/) using the Letter article type, with the full protocol uploaded as an additional file (English language preferred, but we will accept any language), following advice from *Trials*.	Review and approval from the Protocol Editor. Typesetting by production, and publication without copyediting or proofing.

This process ensures rapid publication of a one-page structured summary (based on Consolidated Standards of Reporting Trials for abstracts [4]), which will be indexed by PubMed. The full protocol will be attached as an additional file and assigned a separate DOI (digital object identifier) via Figshare [5]. In the interest of expediting dissemination of this material, the familiar *Trials* formatting and structure will not be necessary for this additional file. This will make full protocols quickly available to other trialists and to trial funders. Registry entries could also link back to the structured summary.

The current pandemic changes everything. For COVID-19 trial protocols, transparency and speed must take precedence. Our editorial processes have been streamlined, and authors will receive direct support from us to ready their structured summary for publication.

We do, however, urge trialists to ensure that their studies are well designed and well reported. Good design and reporting are needed no less in times of pandemics. We also encourage authors to submit their full protocol in the normal way for review to ensure reporting according to the Standard Protocol Items: Recommendations for Interventional Trials guideline [6].

All of us will look back on the COVID-19 pandemic and ask ourselves what we did to help. As a journal dedicated to randomised trials, what *Trials* can do is publish information about COVID-19 trials rapidly, without barriers and without adding to researchers’ workload. This will be the journal’s contribution.

## Data Availability

Not applicable.
